# Introduction of Mature Mast Cells into Bone Marrow Alters Bone Metabolism in Growing Mice

**DOI:** 10.3390/ijms262411952

**Published:** 2025-12-11

**Authors:** Carmen P. Wong, Jessica A. Keune, Kenneth A. Philbrick, Adam J. Branscum, Urszula T. Iwaniec, Russell T. Turner

**Affiliations:** 1 Skeletal Biology Laboratory, School of Nutrition and Public Health, Oregon State University, Corvallis, OR 97331, USA; carmen.wong@oregonstate.edu (C.P.W.);; 2 Biostatistics Program, School of Nutrition and Public Health, Oregon State University, Corvallis, OR 97331, USA; 3 Center for Healthy Aging Research, Oregon State University, Corvallis, OR 97331, USA

**Keywords:** mast cells, bone marrow, gene expression, bone microarchitecture, bone histomorphometry

## Abstract

There is evidence that mast cells contribute to skeletal response to injury, but it is less clear whether these immune cells directly influence normal bone growth and turnover. Mature mast cells are common in the bone marrow of humans and rats, but have not been convincingly demonstrated to be present in the bone marrow of healthy mice, potentially limiting the mouse as a model for characterizing the full range of mast cell/bone cell interactions. An initial goal of this investigation was to comprehensively screen seven strains of mice for mature mast cells in bone marrow. Finding none, we then investigated three approaches to home these cells to the marrow of mice unable to generate mast cells: (1) administration of soluble kit ligand to membrane kit ligand-deficient *Kit^Sl/Sld^* mice, (2) adoptive transfer of wild-type hematopoietic stem cells to kit receptor-deficient *Kit^W/W^*^−*v*^ mice, and (3) adoptive transfer of wild-type mouse bone marrow-derived mast cells generated in vitro and delivered intravenously to *Kit^W/W-v^* mice. Only the third approach was successful. Using this method, we then evaluated the impact of bone marrow-derived mast cells on bone mass, architecture, turnover, and gene expression. The adoptive transfer of mast cells resulted in alterations in cancellous bone microarchitecture and cell populations in the vertebra, and in differential expression of genes associated with bone metabolism in the tibia. Taken together, our results support the concept that bone marrow mast cells influence bone metabolism and suggest that homing mast cells to the bone marrow of mice is a useful model to understand the role of these cells in skeletal health and disease.

## 1. Introduction

Mast cells originate from the myeloid branch of the immune system and play important roles in innate and adaptive immune responses [1]. The cells are found in mammals, birds, reptiles, amphibians, and fish, but the tissue distribution varies among species [2,3]. Mast cell progenitors originate in bone marrow. The immature cells leave the marrow, circulate in the blood, home in many tissues, and, under the influence of the local niche, complete their differentiation to form tissue-specific mature mast cells. Mature mast cells are not found in circulation, suggesting that following maturity, they remain within tissues [4].

The physiological and pathophysiological roles of mast cells have been extensively studied in mast cell-deficient mice, including ones with loss-of-function mutations in the kit receptor or kit ligand [5]. Studies have also been carried out using kit signaling-independent genetic models for mast cell deficiency [6]. As initiators of the inflammatory cascade, there is strong evidence that mast cells play a role in the skeletal response to injury [6]. However, there is model-dependent, contradictory evidence as to the role of mast cells in bone growth and turnover [7]. Specifically, Kroner et al. reported that the ablation of mast cells had no impact on normal skeletal growth or turnover, but mast cells influenced fracture repair and were required for ovariectomy-induced bone loss in mice [8]. In contrast, we reported skeletal changes in growing mast cell-deficient mice but found that mast cell deficiency had no effect on the skeletal response to ovariectomy [9,10,11,12,13,14].

Mast cell differentiation and survival require kit receptor-mediated signaling. The kit receptor (CD 117) is a receptor tyrosine kinase present on the surface of hematopoietic stem cells. Although important in early hematopoietic lineage decision, kit receptor-mediated signaling is generally less important in differentiated hematopoietic lineage cells [15]. Mast cells are unusual, but not unique, in that mature cells express the kit receptor [16]. Importantly, mast cell survival, as well as differentiation, is dependent upon the activation of the kit receptor by the kit ligand (stem cell factor). Fibroblast lineage and endothelial cells produce membrane and soluble forms of the kit ligand [17]. Consequently, mast cells that are present in bone marrow have the potential to interact physically (membrane to membrane) with osteoblasts, preosteoblasts, and bone marrow adipocytes expressing the membrane kit ligand. Additionally, mesenchymal lineage cells may interact at a distance with mast cells through the soluble kit ligand and mast cells with bone cells through many effectors (e.g., cytokines, chemokines, growth factors) that mast cells produce and release [18]. Skeletal phenotypes of mast cell-deficient mice resulting from defects in kit signaling are not uniform across mouse models, indicating that additional factors are at play [9,11]. For example, *Kit^W/W-v^* and *Kit^Sl/Sld^* mice with loss-of-function mutations in the kit receptor and membrane kit ligand, respectively, do not have mature adipocytes in their bone marrow, whereas *Kit^W-sh^* mice, although mast cell-deficient, accrue bone marrow in adipose tissue. The *Kit^W-sh^* mouse has an inversion mutation in the transcriptional regulatory elements upstream of the kit receptor transcription start site on chromosome 5 and has fewer developmental abnormalities attributable to defective kit signaling than the *Kit^W/W-v^* or *Kit^Sl/Sld^* mouse [19].

Kit receptor signaling is important for functions beyond mast cell differentiation, including hematopoiesis, melanocyte survival, and gametogenesis [20]. Loss-of-function mutations resulting in the altered expression of different isoforms of kit ligand or kit receptor differ in their skeletal phenotypes, illustrating the important but complex role of kit signaling in bone metabolism. Our research interests include establishing the role of kit signaling and mast cells in bone tissue. To advance knowledge in how mast cells interact with bone cells, it would be useful to design models in which mature mast cells could be introduced into bone and/or extraskeletal tissues.

Mast cells are normally present within cancellous and endocortical bone compartments in humans and some rodents (e.g., rats). However, as noted, while mast cell precursors originate in bone marrow, the precise stage of differentiation achieved in mice is unknown. We have not detected metachromatic cell granules that are present in mature mast cells in the bone marrow of several commonly investigated mouse strains [21]. There is strong evidence that mature mast cells normally present in bone marrow in other species can interact with preosteoblasts, mature osteoblasts, preosteoclasts, and mature osteoclasts to alter their differentiation and function [21]. The absence of mature mast cells in bone marrow in mice may contraindicate the use of mice as a model to investigate the direct role of mast cells in cancellous and endocortical bone growth and turnover. Therefore, we reviewed histological sections from archived mouse specimens to verify the absence of mature mast cells in marrow. In addition to representative strains of mice, we investigated whether genetic manipulations and interventions that impact bone metabolism localize mast cells to bone marrow in mice. Not finding any mast cells in the bone marrow in the histological sections from representative bones in over 1000 mice, we evaluated the efficacy of several approaches to introduce mast cells into bone marrow. The goal was to develop a model for future investigation of how mast cells in the bone marrow microenvironment interact with resident cell populations. To this end, this study conducted the following: (1) we administered soluble kit ligand to *Kit^Sl/Sld^* mice to induce mast cell differentiation, (2) we adoptively transferred wild-type (WT) hematopoietic stem cells (HSCs) to lethally irradiated *Kit^W/W-v^* mice to restore normal kit signaling, and (3) we differentiated WT mast cells from unfractionated bone marrow in vitro and adoptively transferred these cells into *Kit^W/W-v^* mice. The rationale for the first approach is that the subcutaneous administration of soluble kit ligand to *Kit^Sl/Sld^* mice results in the localization of mast cells to cutaneous connective tissue. As such, it is plausible that mast cells would also localize to bone marrow. Similarly, the adoptive transfer of WT HSCs to *Kit^W/W-v^* mice (approach 2) would be anticipated to lead to an efflux of mast cell precursors from bone marrow to relocate to peripheral connective tissues to complete their differentiation. It is plausible that some of these cells would return to the bone. Finally, adoptively transferred mature mast cells (approach 3) would be anticipated to be quickly cleared from circulation by entry into connective tissues, potentially including bone marrow. Of these three approaches, adoptive transfer of bone marrow-derived mast cells was successful in introducing mast cells into bone marrow. Using this method, we evaluated the impact of mast cells on body composition and on bone architecture, cell populations, and gene expression. We hypothesized that the presence of mast cells in bone marrow would alter bone metabolism.

## 2. Results

### 2.1. Evaluation of Bone Marrow in Mice for Presence/Absence of Mast Cells

**Figure 1:**
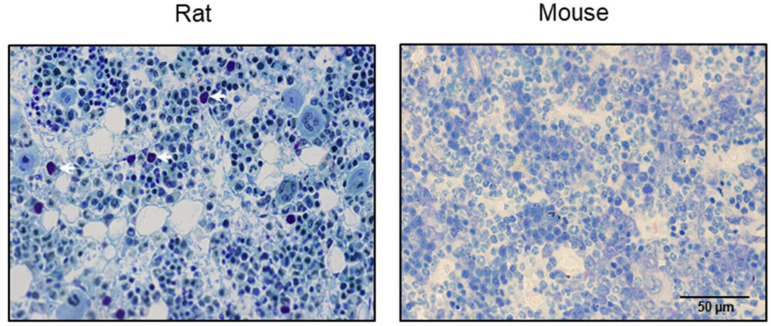
Representative toluidine blue-stained histological section viewed at 64× (scale bar = 50 μm) of proximal tibia metaphysis in Sprague-Dawley rat and distal femur metaphysis in C57BL/6J (B6) mouse showing presence of mature mast cells (exhibiting metachromatic staining) in the rat (positive control) and absence of mature mast cells in the mouse. White arrows point to mast cells. Over 1000 individual histological bone specimens were reviewed for presence of mast cells in mice. Rat histological section is representative of 6-month-old male rats (*n* = 8) with marrow mast cell density of 27 ± 6/mm^2^ (mean ± SE).

As anticipated, mast cells were easily detected in the bone marrow of toluidine blue-stained histological sections of rat, but not mouse, bone (Figure 1). Specifically, mast cells were not detected in the bone marrow in the seven mouse strains evaluated (B6, BALB/cJ, C3H/HeJ, DBA, ICR, Swiss Webster, or WBB6F1/J) (Table 1). Analyses assessing the age, sex, and bone/bone compartment as intrinsic variables revealed no bone marrow mast cells in healthy mice. Similarly, mast cells were not detected in bone marrow following ovariectomy, oophorectomy, high-fat feeding, simulated hyperparathyroidism (continuous infusion of parathyroid hormone (PTH)), simulated microgravity (hindlimb unloading), high-dose irradiation, polyethylene particle-induced systemic bone loss, spinal cord injury-induced systemic bone loss, temperature stress, or hormones, drugs, and genetic manipulations that influence bone metabolism. These null results contrast with the plentiful numbers of mature mast cells routinely observed in the bone marrow of male and female rats [21], and in skin, adipose tissue, and spleen in mice.

### 2.2. Homing Mast Cells to Bone Marrow in Mast Cell-Deficient Mice

***Approach 1: Administration of kit ligand to Kit^Sl/Sld^ mice***. Mast cells were detected in the skin following the administration of soluble kit ligand to *Kit^Sl/Sld^* mice [11]. However, these cells were not detected in the bone marrow (femur) in these mice.

***Approach 2: Adoptive transfer of WT HSCs into Kit^W/W-v^ mice***. Adoptive transfer of WT HSCs into *Kit^W/W-v^* mice following lethal irradiation restored normal kit signaling in the bone marrow [12], but mast cells were not detected in the marrow (femur, lumbar vertebra) in these mice.

**Figure 2:**
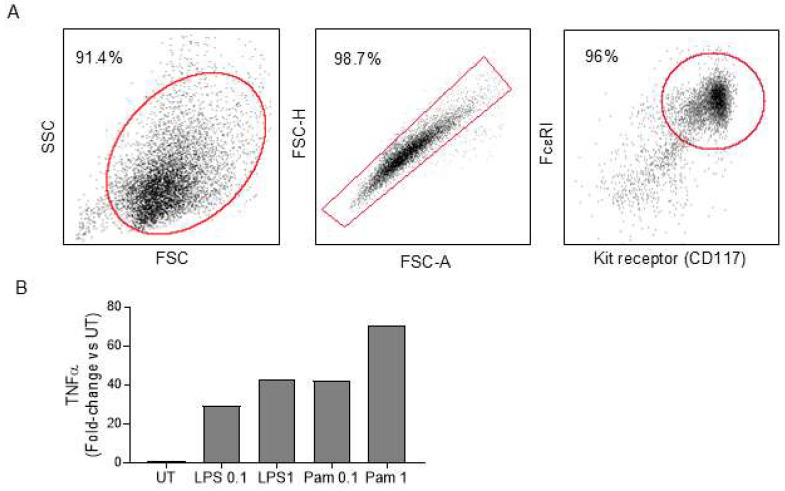
Generation of mast cells. WT bone marrow cells were differentiated ex vivo into mast cells. (**A**) At the end of the 4-week culture, the purity of mast cells was verified by flow cytometry, as determined by the co-expression of kit receptor and FcεR1 on the majority of differentiated cells. The red box/oval denotes the gating strategy of each flow cytometry plot. The percentages shown represent cells within the gated region. A minimum of 10,000 events were acquired and analyzed. (**B**) The ability of cultured mast cells to activate was determined by induced TNFα expression upon TLR2 agonist (Pam3CSK1) and TLR4 agonist (LPS) treatment at 0.1 and 1 µg/mL. TNFα gene expression was determined by real-time PCR and compared relative to untreated (UT) control (*n* = 2/treatment). FSC, forward scatter; SSC, side scatter; FSC-A, FSC area; FSC-H, FSC height.

**Figure 3:**
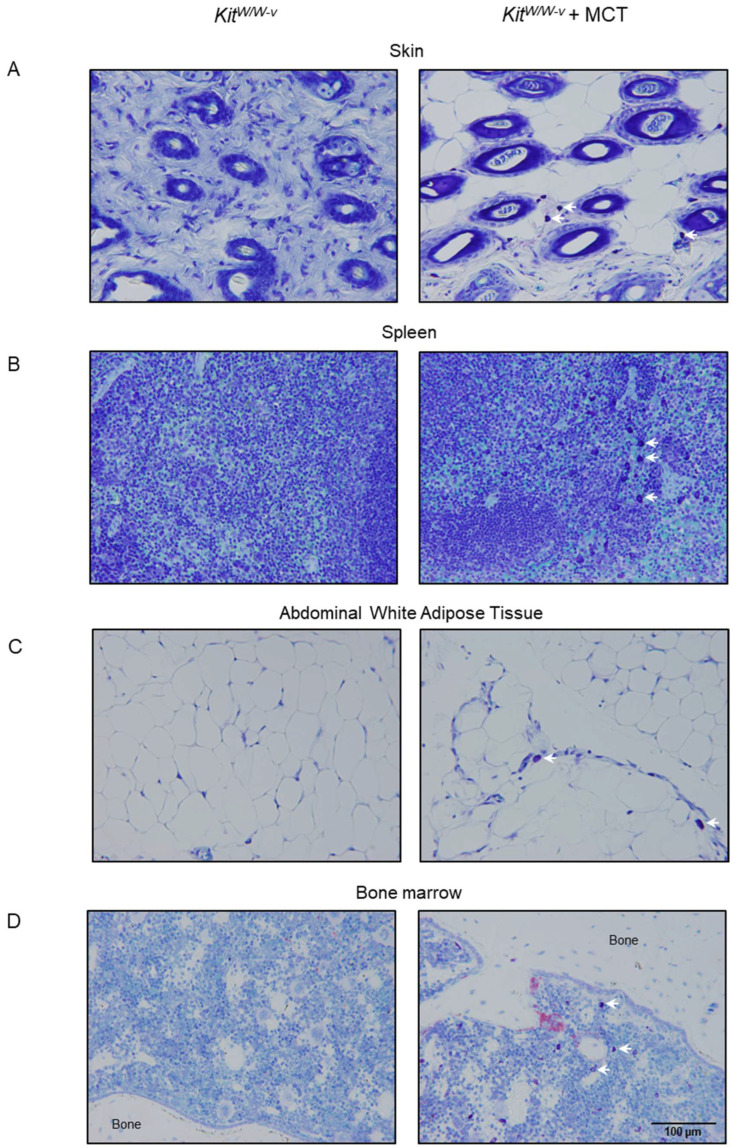
Representative toluidine blue-stained histological sections at 32× (scale bar = 100 μm) of (**A**) skin, (**B**) spleen, (**C**) abdominal white adipose tissue, and (**D**) bone marrow 9 weeks following adoptive transfer of mast cells into *Kit^W/W-v^* mice. White arrows point to mast cells. Vehicle-treated mast cell-deficient *Kit^W/W-v^* mice were used as controls (left column). Quantification of bone marrow mast cells from these mice is presented in Figure 4I.

***Approach 3: Adoptive transfer of WT mast cells into* *Kit^W/W-v^ mice***. Bone marrow cells from WT mice cultured with IL-3 and kit ligand were used to differentiate and expand mast cells count in vitro. Ninety-six percent of these cells co-expressed FcεR1 and CD117, confirming differentiation to the mast cell lineage following 4 weeks in culture (Figure 2A). Incubation of cultured mast cells with toll-like receptor 2 (TLR2) agonist (Pam3CSK1) and TLR4 agonist (LPS) resulted in a dose-dependent increase in TNFα expression, a key inflammatory marker for mast cell activation, indicating functionality in these cells (Figure 2B). Mast cells were common in skin, spleen, white adipose tissue (WAT), and lumbar vertebra following the adoptive transfer of the cultured cells into *Kit^W/W-v^* mice, but were not detected in any of these tissues in untreated *Kit^W/W-v^* mice (Figure 3).

**Body composition and markers of bone turnover**: A adoptive transfer of mast cells had no significant effect on body mass, lean mass, fat mass, percent fat, bone area, bone mineral content, bone mineral density, abdominal WAT weight, uterine weight, blood glucose, or global serum markers of bone turnover, carboxyterminal telopeptide of type 1 collagen (CTX), and osteocalcin (Table 2).

**Figure 4:**
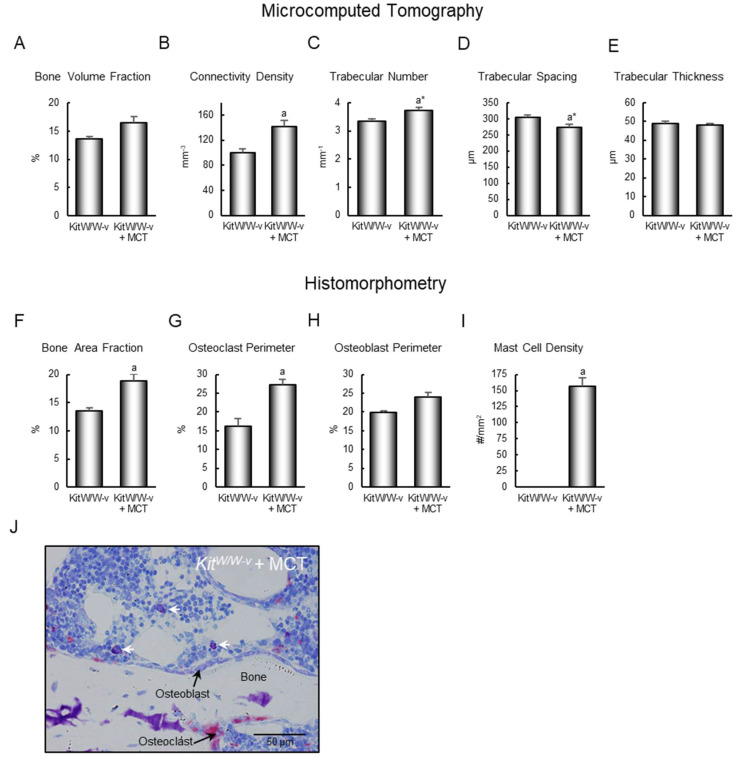
Effect of mast cell transfer (MCT) in *Kit^W/W-v^* mice on (**A**) cancellous bone volume fraction, (**B**) connectivity density, (**C**) trabecular number, (**D**) trabecular spacing, and (**E**) trabecular thickness, and on (**F**) cancellous bone area fraction, (**G**) osteoclast-lined bone perimeter, (**H**) osteoblast-lined bone perimeter, and (**I**) mast cell density in lumbar vertebrae 9 weeks following adoptive transfer of mast cells into *Kit^W/W-v^* mice. Cells measured (64×; scale bar = 50 μm) are shown in panel (**J**), and include osteoclasts, osteoblasts, and mast cells. White arrows point to mast cells. Data are mean ± SE, *n* = 7–8/group. ^a^ Different from control *Kit^W/W-v^*, *p* ≤ 0.05. ^a*^ Different form control *Kit^W/W-v^*, *p* ≤ 0.1.

**Bone architecture (µCT)**: Adoptive transfer of mast cells into *Kit^W/W-v^* mice altered cancellous bone architecture in the lumbar vertebra (Figure 4). Specifically, the adoptive transfer of mast cells resulted in higher connectivity density (Figure 4B), and a tendency (*p* = 0.078) for higher trabecular number (Figure 4C) and lower trabecular spacing (Figure 4D). Significant differences in cancellous bone volume fraction (Figure 4A, *p* = 0.106) or trabecular thickness (Figure 4E) were not detected with treatment.

**Bone histomorphometry**: Adoptive transfer of mast cells resulted in higher bone area fraction (Figure 4F) and osteoclast-lined bone perimeter (Figure 4G). Differences in osteoblast-lined bone perimeter (Figure 4H) were not detected with treatment. Mast cell density increased from 0 to 157 ± 13/mm^2^ (Figure 4I). The cells being assayed are shown in Figure 4J. Bone marrow adipocytes were not observed in the lumbar vertebrae of control *Kit^W/W-v^* mice or *Kit^W/W-v^* mice following adoptive transfer of the WT mast cells.

**Differential gene expression**: We performed 2 PCR gene arrays (osteoporosis and osteogenesis), each evaluating 84 genes. Twenty genes overlapped between the two arrays. Thus, we assayed 144 individual genes related to bone metabolism. Adoptive transfer of mast cells to *Kit^W/W-v^* mice resulted in the differential expression of 40 genes (Table 3). For the 20 genes that overlapped between the two arrays, there were no discrepancies in gene response to adoptive transfer of mast cells. Mast cell-mediated differential gene expression included genes for enzymes (*Alp*, *Phex*), cytokines (*Tgfb3*), receptors (*Bmpr2*, *Casr*, *Cnr2*, *Esr1*, *Essra*, *Fgfr1*, *Itga2*, *Itgb3*, *Lrp5*, *Lrp6*, *Mthfr*, *Tgfbr2*, *Tgfbr3*, *Vdr*), chloride voltage-gated channels (*Clcn7*), vitamin D function (*Dbp*, *Smad3*), skeletal development and maturation (*Serpinh1*, *Ihh*, *Nog*, *Runx2*, *Smad4*, *Smad5*, *Sp7*, *Ahsg*), osteoclast differentiation and function (*Spp1*, *Tnfrsf11a*, *Tnfrsf1b*), structural proteins (*Col14a1*), immune function (*Csf2*, *Tnfaip3*), and angiogenesis (*Vegfa*, *Vegfb*). As expected, gene ontology enrichment analysis showed that the 40 significantly differentially expressed genes were involved in the regulation of ossification, skeletal system development, bone resorption, and osteoblast differentiation (Supplemental Figure S1).

## 3. Discussion

Here we verified that mature mast cells with toluidine blue-stained granules are not normally present in the bone marrow of representative bones of commonly studied inbred and outbred strains of mice. We also showed that neither aging nor interventions that alter bone growth and/or turnover resulted in homing of mature mast cells into bone marrow in mice. Specifically, we evaluated the following interventions that alter bone metabolism: (1) reduction in sex hormone levels (ovariectomy and oophorectomy), (2) hyperparathyroidism, (3) manipulation of diet (e.g., high fat diet), (4) manipulation of skeletal loading (e.g., hindlimb unloading), (5) tissue injury (e.g., high dose radiation, polyethylene particle-induced inflammation, spinal cord injury), (6) temperature stress, (7) hormones (e.g., growth hormone, parathyroid hormone, sex hormones, leptin), (8) drugs (e.g., propranolol, rapamycin), or (9) genetic interventions (e.g., factor 8 KO, TIEG KO, TTP KO, AR TG). As anticipated, mast cells were not detected in the skin, WAT, spleen, or bone marrow of *Kit^W/W-v^* mice. Following subcutaneous administration of soluble kit ligand to *Kit^Sl/Sld^ mice*, mast cells were detected in the skin, but they failed to home to bone marrow. Adoptive transfer of HSCs from WT mice into *Kit^W/W-v^* mice restored kit expression levels in bone marrow to normal, but mast cells were not detected in the marrow. In contrast, mast cells differentiated from bone marrow of WT mice in vitro and adoptively transferred into *Kit^W/W-v^* mice homed to the skin, WAT, spleen, and bone marrow. The presence of mast cells in the bone marrow in these mice was associated with increased cancellous bone area fraction and osteoclast-lined bone perimeter in the lumbar vertebra and altered gene expression in the tibia. The changes in gene expression are consistent with an overall reduction in bone turnover and decreased osteoclast function, as exemplified by lower *Clen7* expression [30].

There is controversy as to whether mast cells, which are commonly found in the intestine and WAT, play a role in energy homeostasis [31,32]. In the present study, adoptive transfer of mature mast cells to mast cell-deficient *Kit^W/W-v^* mice did not influence important measures of body composition, including total weight, lean weight, fat weight, WAT weight, or blood glucose levels. In contrast to our study, which was performed in mice fed a normal diet, studies implicating a role for mast cells in energy homeostasis have been performed in mice fed high-fat diets [33].

The present study was performed on *Kit^W/W-v^* mice. As indicated, these mice have decreased levels of cell-surface Kit receptor. The most consistent skeletal phenotype of *Kit^W/W-v^* mice is the absence of mature bone marrow adipocytes in their long bones and lumbar vertebrae [34]. *Kit^W-sh^* mice are mast cell-deficient but have bone marrow adipocytes [11], and in the present study, adoptive transfer of mast cells into *Kit^W/W-v^* mice resulted in homing of mast cells to bone marrow but did not result in an influx of marrow fat. These observations constitute strong evidence that the absence of bone marrow adipocytes in *Kit^W/W-v^* and *Kit^Sl/Sld^* mice is due to disturbed kit signaling and not to the absence of mast cells. Compared to WT mice, *Kit^W/W-v^* mice exhibit modest differences in bone architecture and turnover [13]. Adoptive transfer of WT HSCs following lethal irradiation into *Kit^W/W-v^* mice restored kit signaling to hematopoietic lineage cells but did not result in homing of mast cells to bone marrow and had minimal effect on bone architecture (femur and lumbar vertebra) [13]. Furthermore, following adoptive transfer of HSCs, there were minimal differences in the expression of genes related to hematopoiesis or bone turnover in the femur between WT and *Kit^W/W-v^* mice [13]. Taken together, these findings strongly support the conclusion that the changes in bone architecture, turnover, and gene expression observed in the present study are due to the introduction of mast cells into the bone marrow.

Systemic mastocytosis can present in a variety of tissues, including skin and bone marrow. Its presence in bone marrow is a rare condition in which mast cells present as compact multifocal aggregates. Gain-of-function mutations of the gene coding for the kit receptor, leading to constitutive signaling through the receptor, are found in >90% of patients with systemic mastocytosis [35]. Osteoporosis is often associated with systemic mastocytosis but not cutaneous mastocytosis [36]. Histologically, bone marrow involvement is typically associated with increased bone turnover, but turnover balance varies [37]. Advanced disease may be associated with osteosclerosis [38]. Systemic mastocytosis with bone loss has a proinflammatory cytokine profile in plasma, whereas mastocytosis with diffuse osteosclerosis shows increased serum/plasma levels of biomarkers related to bone formation and turnover, as well as an immunosuppressive cytokine secretion profile [39]. These findings strongly suggest that mast cell activation in bone marrow and at sites peripheral to bone can have markedly different actions on the skeleton. Additional studies will be required to assess the skeletal impact of normal mast cell function in bone marrow.

In contrast to systemic mastocytosis with a bone marrow component, adoptive transfer of mast cells in the current study resulted in a diffuse distribution of mast cells within the bone marrow. At the gene level, the presence of mast cells was associated with the differential expression of genes related to enzymes, cytokines, receptors, chloride voltage-gated channels, vitamin D function, skeletal development and maturation, osteoclast differentiation and function, immune function, and angiogenesis. This finding suggests that mast cells influence multiple signaling pathways that regulate bone metabolism. At the cellular level, adoptively transferred mast cells homing to bone marrow, while dispersed, were located in the vicinity of bone lining cells, osteoblasts, and osteoclasts, a distribution similar to that observed in normal rats and humans [21]. Mast cell number increases in bone marrow in humans during inflammation, parasitism, uremia, aplastic anemia, select hematologic diseases, and osteoporosis [40,41,42,43,44]. Additional research will be required to establish the role of mast cells in the skeletal response to these pathologies.

Our adoptive transfer of mast cells was limited to females, but we have shown that the skeletal alterations of mast cell-deficient *Kit^W/W-v^* mice in reference to WT mice are similar across sex. As mentioned, mutations in the kit ligand and kit receptor that result in mast cell deficiency have collateral actions that could influence bone metabolism. For example, *Kit^W/W-v^* mice are hypogonadal due to defective gametogenesis [45]. However, we have shown that a deficiency in the kit receptor has minimal effects on the skeletal response to sex hormone deficiency [34].

In the present study, adoptive transfer of mast cells was performed in young growing mice, and mast cells were detected within the skeleton, evaluated 9 weeks later. This finding demonstrates that the stability of mast cells in bone is maintained over time following adoptive transfer. Furthermore, mast cell density in the bone marrow of mice following adoptive transfer was similar to that observed in rats [21]. This suggests that mast cell density within bone marrow following adoptive transfer is physiologically relevant. Additional studies are required to determine the precise time course for the observed changes in cancellous bone balance.

While there are many similarities in bone physiology across vertebrate species, there are also notable differences, some of which may be related to the presence or absence of mature mast cells in bone marrow. For example, parathyroid bone disease in humans is associated with an increase in mast cell density in bone marrow [21]. In rats, simulated hyperparathyroidism recapitulates human parathyroid disease by inducing severe peritrabecular bone marrow fibrosis. The migration of mast cells to bone surfaces, followed by cell degranulation, precedes fibrosis [46]. These responses are inhibited by interventions that interfere with mast cell signaling [21,46]. In contrast to rats, mice are resistant to parathyroid hormone-induced bone marrow fibrosis [21]. Additional research will be required to establish if the absence of mast cells in mice confers resistance to this disease.

In summary, mature mast cells, although common in bone marrow in humans, are not normally present in the bone marrow of mice. This verification should inspire a reevaluation of mouse studies claiming that mast cells play no role in normal bone physiology. Subcutaneous administration of soluble kit ligand into *Kit^Sl/Sld^* mice results in local differentiation of mast cell progenitors to form mature mast cells in skin only, whereas adoptive transfer of purified HSCs into *Kit^W/W-v^* mice restored mast cells to skin, spleen, and adipose tissue. Finally, homing of WT mast cells to the bone marrow of *Kit^W/W-v^* mice by adoptive transfer altered gene expression, bone cell populations, and bone turnover balance. These findings suggest that homing mature mast cells to select tissue locations (e.g., skin only, normal tissue distribution, normal tissue distribution plus bone marrow) is a viable approach for investigating the role of mast cells in mice, residing remotely in peripheral tissues and locally in bone marrow, on bone metabolism. While there are multiple genetic models for mast cell deficiency, we believe this is the first model for introducing mast cells into the bone marrow of mice.

## 4. Methods

### 4.1. Mast Cell Distribution in Skeletal Tissue of Mice

Histological sections of femur, tibia, and/or lumbar vertebra from B6, BALB/cJ, C3H/HeJ, DBA, ICR, Swiss Webster, and WBB6F1/J mice were evaluated for bone marrow mast cells at 20× magnification (Table 1). Toluidine blue is a basic thiazine dye that exhibits metachromasia and detects cytoplasmic granules present in mature mast cells. The regions of interest for histological sections viewed from over 1000 mice were as follows: femur (distal epiphysis, distal metaphysis, diaphysis), tibia (proximal epiphysis, proximal metaphysis, diaphysis), and vertebra (cancellous compartment and endocortical bone perimeter). The total bone marrow area reviewed per histological section ranged from 2 mm^2^ (vertebrae) to 5+ mm^2^ (long bones). Bone marrow cell density in young growing male mice is reported to be ~25,000 cells/mm^2^ [47]. Absence of mast cells in the bone marrow of B6, DBA, and WBB6F1 mice was previously reported [21] and confirmed in the present study. Tibiae from Sprague Dawley rats (Harlan; Indianapolis, IN) were used as positive controls (Figure 1). Additional positive controls consisted of spleen, skin, and adipose tissue from B6 mice fixed for 24 h in 10% buffered formalin and stored in 70% ethanol prior to embedding in paraffin.

It is possible that mast cells home to bone as an adaptive response to treatment. As such, we reviewed toluidine blue-stained histological sections in mice ranging in age from 1 month to 24 months, ovariectomized at the Jackson Laboratory, oophorectomized [27], treated with PTH to simulate hyperparathyroidism [21], fed normal and high-fat diets, hindlimb unloaded [10], subjected to high dose radiation (up to 10 Gy) [13], or experienced systemic bone loss following the insertion of polyethylene particles over calvaria [48], spinal cord injury [49], gene therapy [50], or other interventions (Table 1).

### 4.2. Homing Mast Cells to Bone Marrow in Mast Cell-Deficient Mice

The experimental protocols were approved by the Institutional Animal Care and Use Committee at Oregon State University. All mice were purchased from the Jackson Laboratory (Bar Harbor, Maine) and, unless indicated otherwise, were single-housed at 22 °C on a 12 h light–dark cycle and provided food (rodent chow, Teklad 8604, Harlen Laboratories, Indianapolis, IN, USA) and water ad libitum.

***Approach 1: Administration of kit ligand to Kit^Sl/Sld^ mice***. The purpose of this study was to evaluate whether soluble kit ligand administered to *Kit^Sl/Sld^* mice to induce mast cell differentiation potentiates the homing of mast cells to bone marrow. The experiment has been described in detail [11]. In brief, 5-week-old male mice were divided into 3 treatment groups (*n* = 7–9 mice/group): (1) WT (WBB6F1/J), (2) *Kit^Sl/Sld^* administered carrier (buffered saline), or (3) *Kit^Sl/Sld^* administered secreted kit ligand (mouse recombinant stem cell factor, Sigma, St. Louis, MO). Kit ligand was delivered in saline by daily subcutaneous injection for 3 weeks (100 µg/kg/day, week 1; 150 µg/kg/day, week 2; 200 µg/kg/day, week 3). Following sacrifice, skin and tibia were removed, fixed for 24 h in 10% buffered formalin, and stored in 70% ethanol for histomorphometric analyses for the presence/absence of mast cells.

***Approach 2: Adoptive transfer of WT HSCs into Kit^W/W-v^ mice***. The purpose of this study was to evaluate whether the restoration of normal kit expression levels in the bone marrow of Kit*^W/W-v^* mice results in the presence of mature mast cells in the marrow. The experiment has been described in detail [13]. In brief, 1-month-old female mice were used (*n* = 5–7 mice/group): (1) WBB6F1/J control, (2) *Kit^W/W-v^* control, (3) *Kit^W/W-v^* recipients receiving purified HSCs (HSC → *Kit^W/W-v^*; 5 Gy), and (4) *Kit^W/W-v^* recipients receiving purified HSCs (HSC → *Kit^W/W-v^*, 2 × 5 Gy delivered within a 4 h interval). Following irradiation (^60^Co irradiator source), the mice in groups 3 and 4 were reconstituted with 750 purified WBB6F1/J HSCs by injection (200 µL) in the lateral tail vein. The mice were maintained for 8 weeks subsequent to HSC engraftment. Following sacrifice, tibiae were removed, fixed for 24 h in 10% buffered formalin, and stored in 70% ethanol for histomorphometric analysis of mast cells.

***Approach 3: Adoptive transfer of WT mast cells into* *Kit^W/W-v^ mice***. The purpose of this study was to assess whether the introduction of differentiated WT mast cells into *Kit^W/W-v^* mice results in their homing to the marrow. Four-week-old female *Kit^W/W-v^* mice were randomly divided into two treatment groups (*n* = 7–8 mice/group): (1) vehicle (buffered saline) or (2) mast cell transfer (MCT; mast cells → *Kit^W/W-v^*). Bone marrow cells from the tibia and femur of WT mice were cultured in vitro in RMPI1640 media containing 10% fetal bovine serum, 3 ng/mL interleukin-3, and 15 ng/mL stem cell factor for 4 weeks to differentiate and expand mast cell number. The purity of mast cells was verified by flow cytometry, as determined by the co-expression of kit receptor and FcεR1. The activation of cultured mast cells was evaluated after 24 h stimulation with TLR2 agonist (Pam3CSK1) and TLR4 agonist (LPS) at concentrations of 0.1 and 1 µg/mL. Mast cell activation was determined by real-time PCR, as determined by induced TNFα expression. For the adoptive transfer experiment, mast cells were washed in phosphate-buffered saline (PBS) to remove residual media. The number of healthy live mast cells was determined (>98% viable cells that excluded trypan blue staining), and cells were resuspended in PBS for injections. In total, 6 × 10^6^ mast cells were adoptively transferred via lateral tail vein injections in the mast cell transfer group. The mice were maintained for 9 weeks. Body composition was measured using dual-energy X-ray absorptiometry (DXA). For tissue collection, the mice were anesthetized with isoflurane anesthesia and terminated by decapitation and exsanguination. Abdominal white adipose tissue (WAT) weight (g), uterine weight (g), and blood glucose (mg/dL) were recorded at necropsy. The fifth lumbar vertebrae were removed and placed in formalin for 24 h fixation, then stored at 4 °C in 70% ethanol prior to sequential analysis by microcomputed tomography (μCT) and histomorphometry. Tibiae were removed, flash frozen in liquid N_2_, and stored at −80 °C until processed for RNA analysis. spleens, WAT, and skin were fixed for 24 h in 10% buffered formalin and subsequently embedded in paraffin for histological evaluation.

### 4.3. Dual-Energy X-Ray Absorptiometry

Lean mass and fat mass were measured in vivo using DXA (Piximus, Lunar Corporation, Madison, WI, USA), and % lean mass and % fat mass were calculated. Additionally, total bone area (cm^2^), bone mineral content (g), and bone mineral density (g/cm^2^) were determined. For scanning, the mice were anesthetized with isoflurane anesthesia, positioned on the scanning platform with a nose cone attached to the mouse for isoflurane delivery, and scanned [51].

### 4.4. Micro-Computed Tomography

μCT was used for nondestructive three-dimensional evaluation of bone volume and architecture. The fifth lumbar vertebrae were scanned using a Scanco μCT40 scanner (Scanco Medical AG, Bassersdorf, Switzerland) at a voxel size of 12 µm × 12 µm × 12 µm (55 kVp X-ray voltage, 145 µA intensity, and 200 ms integration time). The filtering parameters sigma and support were set to 0.8 and 1, respectively. The threshold value for evaluation was determined empirically and set at 245 (gray scale, 0–1000).

The entire cancellous bone compartment was evaluated in the vertebral body (154 ± 2 slices; 1848 ± 12 µm). Direct cancellous bone measurements included bone volume fraction (bone volume/tissue volume; volume of total tissue occupied by cancellous bone, %), connectivity density (number of redundant connections per unit volume, 1/mm^3^), trabecular number (number of trabecular intercepts per unit length, 1/mm), trabecular spacing (distance between trabeculae, µm), and trabecular thickness (mean thickness of individual trabeculae, µm).

### 4.5. Histomorphometry

Lumbar vertebrae were dehydrated in graded increases of ethanol and xylene, then embedded undecalcified in methyl methacrylate. Four µm thick sections were cut with a vertical bed microtome (Leica/Jung 2165 Leica Biosystems, Wetzlar, Germany) and affixed to slides with a dried, pre-coated 1% gelatin solution. For cell-based measurements, slides were stained with tartrate-resistant acid phosphatase and counterstained with toluidine blue (Sigma, St. Louis, MO, USA). All data were collected using the OsteoMeasure System (OsteoMetrics, Inc., Atlanta, GA, USA).

The entire cancellous envelope (2.5 ± 0.1 mm^2^) was evaluated in the vertebral body. Static (cell-based) histological measurements included bone area fraction (bone area/tissue area; %), osteoclast perimeter (osteoclast perimeter/bone perimeter; %), osteoblast perimeter (osteoblast perimeter/bone perimeter; %), adipocyte density (#/mm^2^), and mast cell density (#/mm^2^) (Figure 4J). Osteoclast perimeter was determined as a percentage of cancellous bone perimeter covered by multinucleated cells with an acid phosphatase-positive (stained red) cytoplasm. Osteoblast perimeter was determined as a percentage of total bone perimeter lined by plump cuboidal cells located immediately adjacent to a layer of osteoid in direct physical contact with bone. Adipocytes were identified as large circular or oval-shaped cells bordered by a prominent cell membrane, lacking cytoplasmic staining due to alcohol extraction of intracellular lipids during processing. Mast cells were identified by metachromatic staining of histamine-containing granules [52].

The presence or absence of mast cells was also evaluated in paraffin-embedded toluidine blue-stained histological sections of skin, spleen, and WAT.

### 4.6. Gene Expression

Total RNA from the tibia was isolated from 5 to 6 mice/group and individually analyzed. Tibiae were initially pulverized with a mortar and pestle in liquid nitrogen and subsequently in Trizol (Life Technologies, Grand Island, NY, USA). Total RNA was isolated according to the manufacturer’s protocol, and mRNA was reverse-transcribed into cDNA using SuperScript III First-Strand Synthesis SuperMix for qRT-PCR (Life Technologies, Grand Island, NY, USA). The expression of 84 genes related to bone metabolism was determined for the tibia using the mouse Osteoporosis RT^2^ Profiler™ PCR Array (PAMM-170Z) (Qiagen; Carlsbad, CA, USA). The expression of 84 genes related to osteogenic differentiation was determined using the Mouse Osteogenesis RT^2^ Profiler™ PCR Array (Qiagen; Carlsbad, CA, USA). Gene expression was normalized to GAPDH. Relative quantification was determined (ΔΔCt method) using RT^2^ Profiler PCR Array Data Analysis software version 3.5 (Qiagen) after normalization to housekeeping control. Only the significant-averaged fold-change versus control is reported. For TNFα expression in cultured mast cells, RNA was isolated from mast cells after 4 weeks of culture, and cDNA was synthesized as described above. TNFα was detected by real-time PCR, normalized to the expression of Rn18s housekeeping control. The gene list analysis report was generated using Metascape, a web-based portal designed to provide a comprehensive gene list annotation and analysis resource (https://metascape.org/ accessed 11 October 2025).

### 4.7. Statistical Analysis

Data collection was blinded to the group. Mean responses between the control mice and mice injected with WT bone marrow-derived mast cells were compared using independent two-sample *t*-tests or distribution-free Wilcoxon–Mann–Whitney tests. Model diagnostics included the use of the Breusch–Pagan test for homogeneity of variance, plots of residuals versus fitted values, normal quantile plots, and the Anderson–Darling test of normality. The Benjamini and Hochberg [53] method for maintaining the false discovery rate at a maximum of 5% was used to adjust for multiple comparisons. Data analysis was performed using R version 4.4.2.

## Figures and Tables

**Table 1:** Mast cells were not detected in bone marrow of mice across strain, gene alteration, sex, intervention, age, or bone evaluated.

Genetic Background(s)	Gene Alteration(s)	Sex	Intervention	Age at Sacrifice	Bone(s) Evaluated	Source
B6		F	Ovariectomy	14 weeks	LV5	JL
B6		F	Ovariectomy	14 weeks	Femur	JL
B6		F	Particles	13 weeks	Femur	JL
B6		F	Temperature stress, Propranolol	18 weeks	Femur	JL
B6		M	High-fat diet	17 weeks	Femur	JL
B6		M	Spinal cord injury	13 weeks	Femur	JL
B6		M	Temperature stress	16 weeks	LV5, Femur	JL
B6		M, F	Irradiation	10 weeks	LV5	JL
B6	Factor 8 KO, WT	M	None	20 weeks	Femur	[22]
B6	Leptin-deficient (ob/ob), WT	F	Particles	8 weeks	Femur	JL
B6	ob/ob, WT	F	None	8 weeks	Femur	JL
B6	ob/ob, WT	F	Particles	7 weeks	Femur	JL
B6	ob/ob, WT	M	Leptin gene therapy	9 months	Femur	JL
B6	ob/ob, WT	M	Leptin gene therapy, high-fat diet	24, 38 weeks	Femur	JL
B6	ob/ob, WT	M	Hindlimb unloading	18 weeks	Femur	JL
B6	mϕRIP140KD, WT	M	None	9 weeks	Femur	[23]
B6	Tieg KO, WT	M, F	None	8 weeks	Femur	[24]
B6	Tieg KO, WT	F	Ovariectomy	9 weeks	Tibia	[24]
B6	Tieg KO, WT	F	Sclerostin antibody	15 weeks	Femur	[24]
B6	TTP KO, WT	M, F	None	2 years	Femur	[25]
B6, C3H/HeJ		F	Temperature stress	22 weeks	Femur	JL
B6, C3H/HeJ, B6/129	*lit*, LID	F	None	8 weeks	Femur	[26]
B6, DBA, WBB6F1/J		M, F	Continuous PTH (DBA only)	4–26 weeks	Femur	JL
B6, WBB6F1/J		F	Irradiation	12 &16 weeks	Tibia	JL
B6D2F1	AR 2.3-TG, AR 3.6-TG	M	Oophorectomy	25 weeks	LV5	[27]
BALB/cJ		F	Ovariectomy	8 months	Tibia	JL
BALB/cJ		F	2-Methoxyestradiol	9 weeks	Femur	JL
BALB/cJ	Athymic nude	F	Equol, genistein, tumor cells	30 and 35 weeks	Femur	JL
C57BL6/129SvEv	Progesterone receptor KO, WT	F	None	6, 12, 26 weeks	Tibia	[28]
ICR	Nrf2-/-, WT	F	Rapamycin	28–32 weeks	Femur	[29]
Swiss Webster		M	None	9 months		
WBB6F1/J		F	Ovariectomy	8 and 14 weeks	LV5, Tibia	JL
WBB6F1/J		M	Hindlimb unloading, irradiation	18 weeks	Femur	JL

JL (for Jackson Laboratory).

**Table 2:** Effect of mast cell transfer in *Kit^W/W-v^* mice on body composition, blood glucose levels, and serum markers of bone turnover.

	*Kit^W/W-v^*		*Kit^W/W-v^* + Mast Cell Transfer	
**Body composition**				
Body mass (g)	19.9 ± 0.9		20.3 ± 0.9	
Lean mass(g)	15.4 ± 0.7		16.1 ± 0.6	
Fat mass (g)	4.2 ± 0.3		4.0 ± 0.3	
Percent fat	21.3 ± 1.3		19.7 ± 0.8	
Total bone area (cm^2^)	6.98 ± 0.23		7.26 ± 0.17	
Bone mineral content (g)	0.32 ± 0.01		0.33 ± 0.01	
Bone mineral density (g/cm^2^)	0.045 ± 0.001		0.046 ± 0.001	
Abdominal WAT weight (mg)	606 ± 104		588 ± 75	
Uterus weight (g)	42 ± 5		38 ± 6	
**Blood glucose and serum markers of bone turnover**				
Blood glucose (mg/dL)	263 ± 29		258 ± 16	
CTX (ng/mL)	3.8 ± 1.0		5.8 ± 0.9	
Osteocalcin (ng/mL)	95 ± 16		103 ± 11	
Data are mean ± SE. *n* = 7–8/group.				

**Table 3:** Differential expression of genes related to bone turnover in tibia 9 weeks following mast cell transfer (MCT) in *Kit^W/W-v^* versus control *Kit^W/W-v^* mice.

Symbol	Fold Change	*p*-Value
*Ahsg*	−2.0	0.008
*Alpl*	−1.6	0.015
*Bmpr2*	−1.3	0.009
*Casr*	−1.9	0.003
*Clcn7*	−1.7	0.010
*Cnr2*	−2.2	0.002
*Col14a1*	−2.5	<0.001
*Csf2*	−8.5	<0.001
*Dbp*	1.7	0.027
*Esr1*	−1.3	0.008
*Esrra*	−1.4	0.014
*Fgfr1*	−1.2	0.040
*Ihh*	−1.6	0.011
*Itga2*	−1.4	0.013
*Itgb3*	−2.2	0.002
*Lrp5*	−1.6	<0.001
*Lrp6*	−1.4	<0.001
*Mab21l2*	−1.7	<0.001
*Mthfr*	−2.1	0.002
*Nog*	−2.8	<0.001
*Nos3*	−2.2	0.001
*Phex*	−1.2	0.011
*P2rx7*	−1.3	0.028
*Runx2*	−1.5	0.001
*Serpinh1*	−1.9	0.004
*Smad3*	−2.0	0.001
*Smad4*	−1.2	<0.001
*Smad5*	−1.6	0.001
*Sost*	−2.7	<0.001
*Sp7*	−2.5	<0.001
*Spp1*	1.6	0.040
*Tgfb3*	−1.7	0.002
*Tgfbr2*	−1.7	0.003
*Tgfbr3*	−2.1	0.004
*Tnfaip3*	−1.7	<0.001
*Tnfrsf11a*	−2.1	0.001
*Tnfrsf1b*	−1.7	0.001
*Vdr*	−2.3	0.002
*Vegfa*	−1.2	0.008
*Vegfb*	−1.2	0.005

Averaged fold change is calculated as *Kit^W/W-v^* + MCT versus *Kit^W/W-v^*, using the comparative Cq method (2-ΔΔCt method) after normalization via RT^2^ Profiler PCR Array Data Analysis software (Qiagen) version 3.5. Only significant differentially expressed genes are presented (*p* < 0.05). *n* = 5–6/group.

## Data Availability

Data will be made available upon request.

## Supplementary Materials

The following supporting information can be downloaded at: https://www.mdpi.com/article/10.3390/ijms262411952/s1.

## Abbreviations

CTX	carboxyterminal telopeptide of type 1 collagen
DXA	dual energy X-ray absorptiometry
FSC	forward scatter
HSC	hematopoietic stem cell
KO	knockout
LV	lumbar vertebra
MCT	mast cell transfer
μCT	microcomputed tomography
PTH	parathyroid hormone
SSC	side scatter
TLR2	toll-like receptor 2
WAT	white adipose tissue
WT	wild type
