# Supplementary material for: Introduction of Mature Mast Cells into Bone Marrow Alters Bone Metabolism in Growing Mice
Source: Int J Mol Sci. 2025 Dec 11;26(24):11952. doi: 10.3390/ijms262411952 (PMC12733091; doi:10.3390/ijms262411952)
Supplement: Supplementary file 1 [file ijms-26-11952-s001.zip › ijms-3989200-supplementary.pdf]

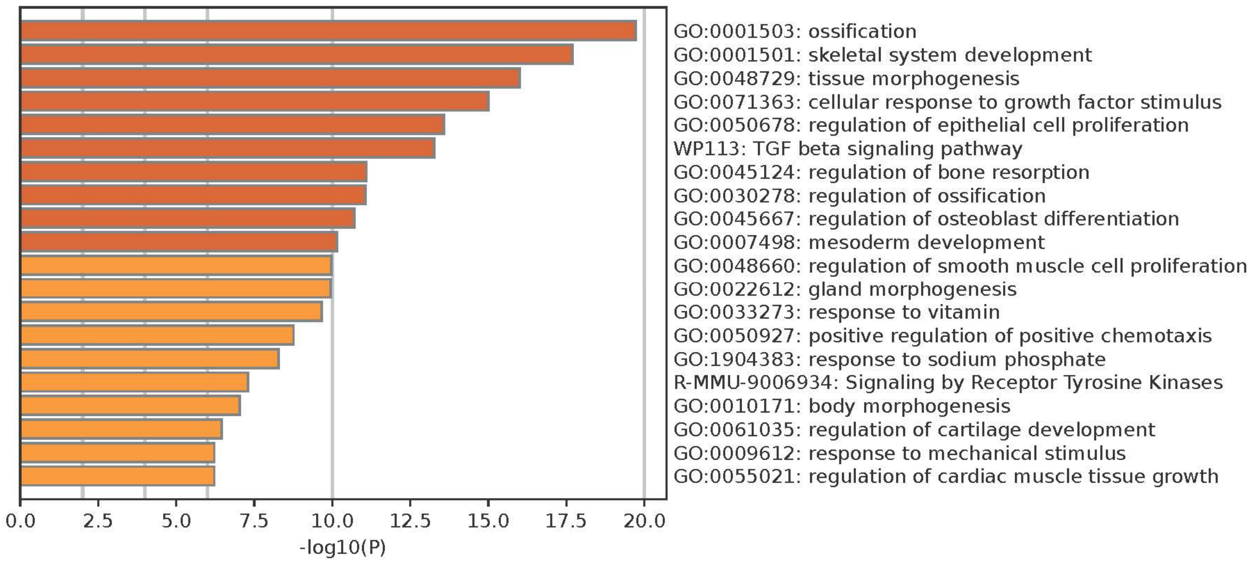

Figure S1. Gene list analysis report. Bar graph of enriched terms across input gene lists, colored by p-values.
